# Effects of varenicline on sympatho-vagal balance and cue reactivity during smoking withdrawal: a randomised placebo-controlled trial

**DOI:** 10.1186/s12971-016-0091-x

**Published:** 2016-08-08

**Authors:** Helge Haarmann, Alexandra Gossler, Peter Herrmann, Slavtcho Bonev, Xuan Phuc Nguyen, Gerd Hasenfuß, Stefan Andreas, Tobias Raupach

**Affiliations:** 1 Department of Cardiology and Pneumology, University Medical Centre Göttingen, D-37099 Göttingen, Germany; 2 Department of Anaesthesiology, University Medical Centre Göttingen, Göttingen, Germany; 3 Mannheim Biomedical Engineering Laboratories, Medical Faculty at Heidelberg University, Mannheim, Germany; 4 Lung Clinic Immenhausen, Immenhausen, Germany; 5 Health Behaviour Research Centre, University College London, London, UK

**Keywords:** Smoking cessation, Sympathetic activity, Baroreflex, Cue reactivity

## Abstract

**Background:**

Varenicline is an effective smoking cessation medication. Some concern has been raised that its use may precipitate adverse cardiovascular events although no patho-physiological mechanism potentially underlying such an effect has been reported. The aim of this study was to test the hypothesis that varenicline impacts on sympatho-vagal balance during smoking withdrawal.

**Methods:**

In this randomised, placebo-controlled trial, muscle sympathetic nerve activity (MSNA), baroreflex sensitivity (BRS), heart rate, and blood pressure were assessed in 17 smokers four weeks before a quit attempt (baseline) and again on the third day of that quit attempt (acute smoking withdrawal).

**Results:**

Regarding the primary endpoint of our study, we did not find a significant effect of varenicline compared to placebo on changes in MSNA burst incidence between baseline and acute smoking withdrawal (−3.0 ± 3.3 vs.−3.9 ± 5.0 bursts/100 heart beats; *p* = 0.308). However, heart rate and systolic blood pressure significantly decreased in the placebo group only, while no significant changes in these parameters were observed in the varenicline group. Exposure to smoking cues during acute withdrawal lead to a significant increase of heart rate in the placebo group, while heart rate decreased in the varenicline group, and the difference in these changes was significant between groups (+2.7 ± 1.0 vs.−1.8 ± 0.5 1/min; *p* = 0.002). In all 17 participants combined, a significant increase in heart rate during smoking cue exposure was detected in subjects who relapsed in the course of six weeks after the quit date compared to those who stayed abstinent (+2.5 ± 1.2 vs.−1.1 ± 0.7; *p* = 0.018). Six-week abstinence rates were higher in the varenicline group compared to placebo (88 vs. 22 % *p* = 0.015).

**Conclusion:**

We did not find evidence of adverse effects of varenicline on sympatho-vagal balance. Varenicline probably blunts the heart rate response to smoking cues, which may be linked to improved cessation outcome.

## Background

Among all first-line smoking cessation drugs currently available, varenicline is particularly effective: In a recent network meta-analysis, it was found to produce higher abstinence rates than placebo, bupropion, or single forms of nicotine replacement therapy [1]. Despite one large randomised trial showing no increased risk of cardiovascular events in smokers taking varenicline compared to placebo [2], a meta-analysis of 14 trials published before March 2011 concluded that varenicline use was associated with a 1.06 % risk of adverse cardiovascular events while this risk was only 0.82 % in patients taking placebo, and this difference was significant [3]. So far, no plausible pathophysiological mechanism underlying this effect has been suggested.

Research of the past two decades has identified disturbances of the autonomic nervous system as key mechanisms involved in the pathophysiology of cardiovascular diseases including heart failure [4], coronary heart disease [5], and hypertension [6, 7]. In these conditions, a shift of sympatho-vagal balance towards increased sympathetic activity has been observed. This may play a major role in promoting disease progression: In patients suffering from heart failure, increased sympathetic activity is directly linked to worse clinical outcome [8].

In 1998, Narkiewicz and colleagues demonstrated that acute smoking elicited a significant increase in sympathetic activity [9]. In addition, smoking reduces vagal modulation of the sinuatrial node [10–12] and baroreflex gain [13], thus further contributing to sympatho-vagal imbalance. Given the link between sympatho-vagal imbalance and cardiovascular morbidity and mortality, some of the health benefits of quitting smoking may be attributable to decreased sympathetic activity and improved vagal function following smoking cessation. In fact, increased heart rate variability (indicative of improved vagal function) has recently been documented even after only three days of abstinence [14]. This suggests that sympathetic activity is probably reduced during acute smoking withdrawal; however, we are not aware of any studies assessing sympathetic activity during withdrawal.

So far, the effects of varenicline on sympathetic and vagal activity have not been investigated. As nicotinic acetylcholine receptors are involved in signal transduction within the sympathetic nervous system, the partial receptor agonist varenicline may induce sympatho-excitation, thereby offsetting some of the beneficial effects of smoking cessation on sympatho-vagal balance. At the same time, the acute stress reaction associated with cravings (e.g. upon exposure to smoking cues [15, 16]) might be blunted in the presence of varenicline due to its partially agonistic action that is believed to relieve craving. Moreover, therapeutic doses of varenicline desensitize the nicotine receptor. Receptor desensitization may also play a role in blunting the stress response associated with cravings, and may attenuate the impact of inhaled nicotine during smoking relapse [17].

The first aim of this study was to investigate the effect of varenicline on sympatho-vagal balance during smoking withdrawal. We hypothesised that acute (48–72 h) smoking withdrawal elicits a decrease in sympathetic activity and that varenicline attenuates this effect.

Our second aim was to investigate the effect of varenicline on changes in sympatho-vagal balance elicited by exposure to smoking cues during acute smoking withdrawal. We hypothesised that varenicline would impact on sympatho-vagal balance during smoking cue exposure, and that the direction and magnitude of changes elicited by smoking cues would be related to short-term cessation rates.

## Methods

The study was registered with BfArM, the European Clinical Trials Database (EudraCT No. 2011-000843-26), and clinicaltrials.gov (NCT01474265) and approved by the Ethics Committee of Göttingen University (Application number 29/4/11). At the screening visit, eligibility of the subjects was assessed and written informed consent obtained.

Male and female smokers aged between 25 and 60 years and displaying at least moderate nicotine dependence (≥5 points on the Fagerström Test of Nicotine Dependence; FTND [18]) were invited to participate in the study. Pregnant women and subjects suffering from diseases known to increase sympathetic activity (e.g., heart failure, COPD, depression, obstructive sleep apnoea, pulmonary-arterial hypertension) or taking drugs impacting on sympatho-vagal balance (e.g., beta blockers, selective serotonin reuptake inhibitors, theophylline) were excluded.

### Study outline

**Fig. 1:**
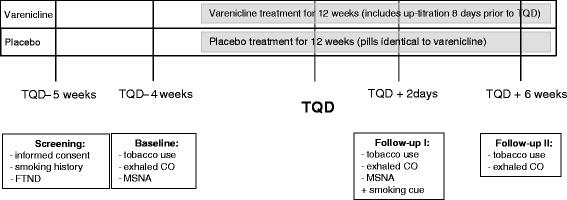
Study timeline

The timeline of the study is displayed in Fig. 1. At the baseline visit, a smoking history was taken, and baseline sympatho-vagal balance was measured as described below. Following the baseline visit, participants were randomised to either the varenicline or the placebo group. All subjects received professional smoking cessation support in a six-week group programme with proven effectiveness [19]. Each of the weekly sessions lasted 90–120 min, with a maximum of 12 participants per group. During the first sessions, medical and psychological issues were discussed, and participants were asked to observe their own smoking behaviour. During the remainder of the course, coping skills and relapse prevention were addressed.

Participants were asked to set their target quit date (TQD) between the second and the third course session and instructed to start using their study medication eight days before their TQD. Study medication was up-titrated as previously described for varenicline [20]. A second evaluation of sympatho-vagal balance was scheduled for day 2 of the quit attempt (follow up I) as withdrawal symptoms tend to peak around 2–3 days after the quit date [21]. Short-term continuous abstinence was assessed during a third visit six weeks after the TQD (follow up II). Abstinence at both follow up points was biochemically validated (exhaled carbon monoxide (CO) concentration of < 6 ppm). Subjects failing to provide a CO reading were assumed to be smoking.

### Experimental protocol

The same experimental protocol was used for both assessments of sympatho-vagal balance (baseline & follow up I). Smokers had to abstain from cigarette smoking 1 h before baseline. Subjects were in a supine position. First, continuous recordings of heart rate and blood pressure (non-invasive beat-to-beat analysis using the Portapres device; FMS®, Amsterdam, Netherlands) were established.

After mapping the course of the peroneal nerve around the head of the fibula by transcutaneous electrical stimulation (Stimuplex HNS 11, B Braun, Melsungen, Germany), a tungsten microelectrode (shaft diameter 200 μm and tip of 1–5 μm, FHC, Bowdoin, ME, USA) was inserted into the nerve. Nerve signals were amplified (~100,000 times), filtered (band width of 700–2000 Hz), and processed by a resistance-capacitance integrating network with a time constant of 0.1 s, providing a mean voltage display of muscle sympathetic nerve activity (MSNA) (Nerve Traffic Analysis System, model 662C-3, University of Iowa, Iowa City, USA). The procedure and the criteria for obtaining a satisfactory recording of MSNA have been described previously [22].

At baseline, MSNA, blood pressure and heart rate were continuously recorded for 20 min at rest. At follow up I, recordings under resting conditions were repeated, and following that, participants were exposed to a smoking cue: They were allowed to touch a pack of their favourite cigarette brand and their lighter for two minutes.

### Data analysis

Two minutes of raw data were collected for each of the two conditions (rest and smoking cue exposure). MSNA was assessed by quantifying grouped single fibre activity. These ‘bursts’ of sympathetic activity were identified by inspection of the mean voltage neurogram, and the number of bursts observed during the two-minute period were reported as burst frequency (bursts/min) or as burst incidence (bursts/100 heart beats). Baroreflex sensitivity (BRS), reflecting vagal activity, was calculated using the sequence method [23]. Briefly, recordings of continuous systolic blood pressure measurements were analyzed for sequences of ≥ 4 consecutive beats with progressive increase or decrease in blood pressure and a linearly related change in the pulse interval (correlation coefficient r ≥ 0.85). BRS (ms/mmHg) was identified as the slope of the regression line between pulse interval and blood pressure change within each sequence.

Statistical analyses were performed with SPSS Statistics 21 (IBM Corporation, New York, USA). The primary endpoint was the between-group difference in changes in MSNA burst incidence (bursts/100 heart beats) between baseline and follow up I. These were assessed by a repeated measures ANOVA. Within-group changes were assessed by paired T tests. Between-group differences at baseline were analysed by unpaired T tests. Abstinence rates in the placebo and the varenicline group were compared using Fisher’s exact test. Data are presented as mean ± SEM (continuous variables), as appropriate. Significance levels were set to 0.05.

Based on the results obtained in a previous study [24], complete MSNA data from 12 participants were needed in order to detect a change by 7 bursts/100 heart beats on an alpha level of 5 with 80 % power. In order to account for drop-outs due to limited MSNA signal quality (30 % of cases), a total of 17 subjects had to be enrolled in each group.

## Results

A total of 36 Caucasian smokers were enrolled in the study, but 12 were excluded due to early relapse (eight in the placebo and four in the varenicline group). Another seven subjects were excluded from the analysis due to the fact that an MSNA signal could not be obtained on both baseline and follow up I to assess the primary endpoint (two in the placebo and five in the varenicline group). Subjects that failed to provide a CO reading (three at follow up I; two at follow up II) were assumed to be smoking.

Table 1 presents baseline characteristics of those 17 subjects that were included in the analysis. These subjects had stopped smoking on their target quit date and remained continuously abstinent until follow up I. With an average FTND score of 6.8 ± 0.4 points in the overall group, participants were moderately dependent. There were no significant differences between the groups with regard to baseline characteristics (Table 1).

**Table 1:** Subject characteristics at baseline

	Placebo	Varenicline	p (independent T test)
	*n* = 9	*n* = 8	
Female gender	56 % (5)	50 % (4)	0.488
Age [years]	42.4 ± 3.6	44.5 ± 3.8	0.699
Height [cm]	174.0 ± 2.1	176.1 ± 4.1	0.641
Weight [kg]	82.8 ± 4.8	83.1 ± 4.8	0.960
Body Mass Index [kg/m^2^]	27.3 ± 1.4	26.8 ± 0.9	0.775
Number of cigarettes smoked per day	22.3 ± 2.4	26.9 ± 1.7	0.151
Age at onset of smoking	15.4 ± 0.5	14.6 ± 0.4	0.220
Pack Years	24.1 ± 3.4	37.9 ± 6.3	0.066
number of previous quit attempts	3.6 ± 1.5	3.1 ± 1.2	0.840
FTND score	6.3 ± 0.5	7.4 ± 0.5	0.162
MSNA burst frequency [bursts/min]	37.8 ± 5.3	37.4 ± 2.8	0.950
MSNA burst incidence [bursts/100 heart beats]	58.1 ± 7.1	56.9 ± 4.6	0.890
Heart rate [1/min]	63.8 ± 2.4	66.4 ± 3.3	0.529
BRS [ms/mmHg]	15.8 ± 6.9	9.4 ± 2.5	0.454
SBP [mmHg]	136.4 ± 4.1	130.8 ± 4.3	0.354
DBP [mmHg]	80.6 ± 2.6	83.3 ± 2.7	0.481

Changes in parameters between baseline and follow up I are displayed in Table 2. Regarding the primary endpoint of our study, changes in MSNA burst incidence (bursts/100 heart beats) did not differ between groups. During smoking withdrawal, MSNA as expressed in burst frequency (bursts/min), as well as heart rate and systolic blood pressure, significantly decreased in the placebo group only, as assessed by paired T tests. In the varenicline group there were no significant changes during smoking withdrawal. As groups were compared, no significant group differences in changes were found. The significant decrease in MSNA burst frequency, heart rate and systolic blood pressure, which was observed in the placebo group, was accompanied by a tendential increase in BRS.

**Table 2:** Changes (Δ) of parameters at rest between baseline and acute smoking withdrawal (follow up I)

Parameters	Δ (follow up I - baseline)		*p* value for interaction (repeated measures ANOVA)
	Placebo	Varenicline	
MSNA burst frequency [bursts/min]	−6.3 ± 2.4*	−2.7 ± 2.4	0.308
MSNA burst incidence [bursts/100 heart beats]	−3.0 ± 3.3	−3.9 ± 5.0	0.886
Heart rate [1/min]	−6.9 ± 2.6*	−0.5 ± 2.1	0.078
BRS [ms/mmHg]	+6.6 ± 3.4	−2.3 ± 2.9	0.086
SBP [mmHg]	−8.4 ± 3.4*	−3.4 ± 2.5	0.277
DBP [mmHg]	−3.4 ± 2.3	+0.4 ± 3.3	0.333

*p < 0.05 for within-group changes assessed in a paired T test

Changes in parameters between resting conditions at follow up I and exposure to smoking cues at follow up I are displayed in Table 3. There were no significant changes in MSNA, BRS, or blood pressure within groups and changes did not differ between groups for these parameters. Heart rate significantly increased during smoking cues in the placebo group. In contrast, heart rate significantly decreased in the varenicline group. The difference of these changes between groups was significant.

**Table 3:** Changes (Δ) of parameters during acute withdrawal (follow up I) between rest and smoking cue exposure

Parameters	Δ (smoking cues – rest)		*p* value for interaction (repeated measures ANOVA)
	Placebo	Varenicline	
MSNA burst frequency [bursts/min]	−0.7 ± 1.4	+0.3 ± 2.4	0.747
MSNA burst incidence [bursts/100 heart beats]	−3.5 ± 2.5	+1.6 ± 3.6	0.262
Heart rate [1/min]	+2.7 ± 1.0*	−1.8 ± 0.5*	0.002
BRS [ms/mmHg]	−3.7 ± 2.0	+11.4 ± 7.5	0.061
SBP [mmHg]	+2.3 ± 2.6	+4.0 ± 2.4	0.639
DBP [mmHg]	+2.5 ± 1.7	−1.5 ± 2.9	0.228

*p < 0.05 for within-group changes assessed in a paired T test

Two out of nine subjects in the placebo and seven out of eight subjects in the varenicline group remained continuously abstinent over a period of six weeks after the quit date (22 vs. 88 %; *p* = 0.015). Table 4 displays the association between changes in parameters during exposure to smoking cues and smoking abstinence or relapse. The overall group was divided into two subgroups of subjects that were continuously abstinent and those who relapsed. No significant between-group differences in pre-post changes were observed for MSNA, BRS, or blood pressure. In subjects who remained abstinent, heart rate decreased during cue exposure, while it increased in subjects who went on to relapse. While both within-group changes were non-significant, a repeated-measures ANOVA revealed a significant interaction between group and cue exposure.

**Table 4:** Association between changes (Δ) in parameters following exposure to smoking cues during withdrawal (follow up I) and continuous abstinence at follow up II (six weeks after the target quit date)

Parameters	Δ (smoking cues – rest)		*p* value for interaction (repeated measures ANOVA)
	Continuous abstinence (*n* = 9)	Relapse (*n* = 8)	
MSNA burst frequency [bursts/min]	+0.5 ± 2.1	−1.1 ± 1.6	0.554
MSNA burst incidence [bursts/100 heart beats]	+1.4 ± 3.2	−4.0 ± 2.8	0.237
Heart rate [1/min]	−1.1 ± 0.7	+2.5 ± 1.2	0.018
BRS [ms/mmHg]	+9.3 ± 6.6	−3.8 ± 2.3	0.107
SBP [mmHg]	+4.5 ± 2.2	+1.0 ± 2.9	0.342
DBP [mmHg]	+0.4 ± 2.5	+1.3 ± 2.0	0.779

## Discussion

We conducted a randomised trial comparing the effects of varenicline and placebo on sympatho-vagal balance and cue reactivity during acute smoking withdrawal. Regarding the primary endpoint of our study, we did not find a significant effect of varenicline versus placebo on changes in sympathetic activity as expressed in MSNA burst incidence between baseline and acute smoking withdrawal.

To our knowledge, this is the first study to measure MSNA during acute smoking withdrawal. We found a significant decrease in heart rate accompanied by a decrease in MSNA burst frequency during withdrawal in subjects treated with placebo only (Table 2). These findings are in line with earlier findings of decreased sympathetic activity [25] and increased vagal activity [14, 26] following smoking cessation. Likewise, our data corroborate previous reports of a significant decrease in heart rate and systolic blood pressure during withdrawal [27]. Numerous studies have reported a decrease in resting heart rate during acute withdrawal [28], and one common explanation for this finding is that nicotine stimulates norepinephrine release [29] and might even have direct effects on cardiac tissue [30]. Given that significant changes in heart rate, systolic blood pressure and burst frequency were only found in the placebo group, we assume that varenicline might have blunted the mechanisms underlying these effects due to its partially agonistic action at nicotine receptors.

Regarding the second aim of our study, we assessed the effect of varenicline on smoking cue reactivity during withdrawal (Table 3). We detected a significant effect of varenicline on the heart rate response towards smoking cues. While heart rate increased in the placebo group, it decreased in the varenicline group. There was a tendential difference (*p* = 0.061) between groups regarding the change in BRS, which leaves open the question, if these changes in heart rate were regulated by changes in BRS.

With regards to continuous smoking abstinence over the course of six weeks after the quit date, we found a significant difference in the heart rate response towards smoking cues between subjects that remained abstinent and those who went on to relapse (Table 4). A larger increase in heart rate following cue exposure was noted in subjects who relapsed. As discussed above, varenicline might have blunted the increase in heart rate or even reduced the heart rate in response to smoking cues. This might be a result of partial nicotine receptor agonism and/or receptor desensitisation elicited by varenicline. Relieving craving and blunting the stress response towards smoking cues might be one of the reasons why varenicline is an effective smoking cessation agent. Our study supports this assumption on a pathophysiological level.

The association between cue-induced changes in heart rate and subsequent quitting success has not been studied in great detail, and the available studies took different experimental approaches than the one used in the present trial [31, 32]. If our findings are replicated in adequately powered trials, assessment of cue-induced heart rate changes during withdrawal might be introduced as a simple tool to predict the likelihood of relapse. Given that heart rate can be modulated by simple interventions such as slow breathing [24], this approach might then even pave the way for new interventions fostering abstinence on the subconscious level [33].

### Strengths and limitations

The results presented here were derived from a randomised-controlled trial using sophisticated experimental methods. In addition to being highly reproducible, MSNA measurements provide a clearer and more consistent reflection of acute and chronic changes in sympathetic activity than other measures of sympathetic activity [34–37]. Smoking status was biochemically validated, and we took a conservative approach by treating all subjects with missing data on their smoking status as smokers [38]. Interpretation of our findings is limited by the fact that subgroup analyses involved small samples. We failed to detect a significant effect on the primary endpoint of the study, and as the power calculation was based on a sample size of 12 in both groups, the study was slightly underpowered. Finally, we did not assess reactivity to neutral cues that are unrelated to smoking. As recent research suggests that cue-induced changes in some measures of vagal tone only occur following smoking cues (as opposed to neutral cues) [39], future studies assessing sympatho-vagal tone during withdrawal should also include neutral cues.

## Conclusions

This study does not suggest any adverse effects of varenicline on sympatho-vagal balance, which is in line with recent reviews that affirmed cardiovascular safety of varenicline [40–43]. Varenicline probably blunts the heart rate response to smoking cues, which may be linked to improved cessation outcome.

## Abbreviations

BRS: Baroreflex sensitivity
CO: (Exhaled) carbon monoxide
COPD: Chronic obstructive pulmonary disease
DBP: Diastolic blood pressure
FTND: Fagerström Test of Nicotine Dependence
MSNA: Muscle sympathetic nerve activity
SBP: Systolic blood pressure
TQD: Target quit date
